# Blood cytokine expression correlates with early multi-organ damage in a mouse model of moderate hypothermia with circulatory arrest using cardiopulmonary bypass

**DOI:** 10.1371/journal.pone.0205437

**Published:** 2018-10-11

**Authors:** Ruslan Natanov, Faikah Gueler, Christine S. Falk, Christian Kühn, Ulrich Maus, Erin C. Boyle, Thierry Siemeni, Ann-Katrin Knoefel, Serghei Cebotari, Axel Haverich, Nodir Madrahimov

**Affiliations:** 1 Department of Cardiothoracic, Transplantation and Vascular Surgery, Hannover Medical School, Hannover, Lower Saxony, Germany; 2 Department of Nephrology, Hannover Medical School, Hannover, Lower Saxony, Germany; 3 Institute of Transplant Immunology, Integrated Research and Treatment Center Transplantation (IFB-Tx), Hannover Medical School, Hannover, Lower Saxony, Germany; 4 Department of Pneumology, Hannover Medical School, Hannover, Lower Saxony, Germany; National Institutes of Health, UNITED STATES

## Abstract

Cardiopulmonary bypass (CPB) with moderate hypothermic cardiac arrest (MHCA) is essential for prolonged complex procedures in cardiac surgery and is associated with postoperative complications. Although cytokine release provoked through MHCA under CPB plays a pivotal role in postoperative organ damage, the pathomechanisms are unclear. Here, we investigated the cytokine release pattern and histological organ damage after MHCA using a recently described mouse CPB model. Eight BALB/c mice underwent 60 minutes of circulatory arrest under CPB, were successively rewarmed and reperfused. Blood cytokine concentrations and liver and kidney function parameters were measured and histological changes to these organs were compared to control animals. Our results showed a marked increase in proinflammatory cytokines and histological changes in the kidney, lung, and liver after CPB. Furthermore, clinical chemistry showed signs of hemolysis and acute kidney injury. These results suggest early onset of solid organ injury which correlates with increased leukocyte infiltration. A better understanding of the interplay between pro-inflammatory cytokine activation and solid organ injury in this model of CBP with MHCA will inform strategies to reduce organ damage during cardiac surgeries in the clinic.

## Introduction

Cardiopulmonary bypass (CPB) is an essential component in major cardiac surgery for patients suffering from cardiopulmonary failure. During CPB, blood is circulated and oxygenated by the heart-lung machine (HLM) before it is returned to the patient. An inflammatory response occurs during this process due to blood contact with the large foreign surface of the CPB tubing system. Circulating inflammatory cytokines are significantly higher in patients undergoing CPB in comparison to off-pump coronary artery bypass (OPCAB) [1,2].

Due to the need for prolonged CPB in complex cardiac surgeries, moderate hypothermic circulatory arrest (MHCA) has become a common clinical procedure. Complications after MHCA including pulmonary, renal, and myocardial dysfunction, significantly contribute to impaired postoperative outcomes and were shown to be partially mediated through cytokine release during MHCA [3–6]. Interestingly, while hypothermia alone does not alter the release of systemic inflammatory mediators, MHCA causes enhanced pulmonary dysfunction (higher percentage of lung water, worse alveolar to arterial oxygen gradient, and greater pulmonary vascular resistance) as well as enhanced myocardial dysfunction (higher right ventricular and pulmonary pressure, worse cardiac index and lower blood pressure) compared to total circulatory arrest alone [3,7]. Release of systemic inflammatory mediators can initiate an inflammatory cascade but the contribution of innate and adaptive immune responses has not been studied in detail. In severe cases, this inflammatory activation can lead to a systemic inflammatory response syndrome (SIRS), multi-organ failure (MOF), and death [8]. Several inflammatory cytokines, including the pro-inflammatory cytokine interleukin-6 (IL-6) and the anti-inflammatory cytokine interleukin-10 (IL-10), have been measured in high concentrations after CPB and MHCA. Both cytokines are thought to play important roles in the inflammatory response and solid organ injury following CPB [9–14].

Histologically, signs of acute kidney injury (AKI) with increased renal tubular dilation, interstitial edema, and inflammatory cell infiltration after MHCA have been described [15]. Furthermore, MHCA leads to acute lung injury with thickening of the alveolar wall, micro-thrombosis in the pulmonary capillaries, and interstitial edema [16]. Although some MHCA research has been done in large animal models, there are certain disadvantages to these models due to their high costs and impracticability. To our knowledge, there is no published literature describing cytokine expression patterns in combination with histological changes after MHCA in a small animal model. In this study, we employed our novel recently-described mouse model of CPB [17] to assess the cytokine response and its correlation with early histological changes and acute multi-organ injury after MHCA.

## Material and methods

### Animals

A total of 16 male BALB/c mice were purchased from Charles River. Eight 12 weeks old animals weighing between 25–35 g were used for CPB. The remaining animals were used as healthy controls. This study was conducted in compliance with the German Animal Protection Law. The animal protection committee of the local authorities [Lower Saxony State Department for Food Safety and Animal Welfare (LAVES)] approved all experiments (Approval: 33.12-42502-04-14/1556).

### Surgical procedure and cardiopulmonary bypass

The surgical procedure and CPB were carried out as described previously [17]. In brief, all animals were anesthetized with isoflurane and orotracheally intubated. Additional analgesia was given by subcutaneous injection of Carprofen (Zoetis, Parsippany, NJ, USA) at a dose of 5mg/kg body weight.

A midline skin incision was made to the neck, exposing the right jugular vein and left carotid artery. Subsequently, a 5 mm long upper sternotomy incision was made, exposing the aortic arch. For better exposition, a loop (8–0 silk) was placed around the ascending aorta and pulled cranially to facilitate aortic clamping.

Prior to venous and arterial cannulation, priming of the CPB circuit was performed using 850 ml of a 3:1 solution of Tetraspan: Sterofundin (B Braun Medical, Melsungen, Hesse, Germany) that had been heparinized with 30 IU/ml of perfusion solution. Buffering of the solution was carried out using 2.5% v/v of an 8.4% solution of sodium bicarbonate. An arterial pressure line was used for blood sampling and blood pressure monitoring. After initiation of stable CPB, respiratory arrest was maintained for 60 minutes and moderate hypothermia was initiated by bringing the body temperature to 28°C.

Cardioplegia was induced by perfusion with 7.45% KCL solution and circulatory arrest was maintained for 30 minutes. After 30 minutes of circulatory arrest, re-ventilation began and the animal was rewarmed to 37°C. Cardioplegia was confirmed both visually and electrocardiographically. The aortic clamp was removed from the ascending aorta allowing for heart reperfusion. During the experiment, blood gas analysis was performed at four time points to evaluate the hemodynamic, respiratory, and acid-base status. After termination of CPB, animals were monitored for another 20 min and then sacrificed by exsanguination under ongoing cardiac arrest and deep isoflurane anesthesia. Blood and organs were harvested for further analysis.

### Blood gas analysis

Blood gas analysis (BGA) from blood obtained from the femoral artery was done to evaluate the oxygenation and metabolic state during CPB. Initial pO2 values showed 135 mmHg– 590 mmHg. After 15 minutes of respiratory arrest, oxygen saturation ranged between 82 mmHg– 506 mmHg. Best results were achieved with an oxygen/air mixture at FiO2 80%.

### Liver and renal function assessment

To evaluate liver and renal function after CPB, clinical chemistry was carried out. Renal function was evaluated by serum creatinine and blood urea nitrogen (BUN). Liver function was analyzed using alanine transaminase (ALT) and aspartate transaminase (AST). All measurements were done on an AU 400 Olympus analyzer according to the manufacturer´s instruction.

### Quantification of plasma cytokines and chemokines

Heparinized mouse blood was collected, centrifuged for 4 min at 3000 g, and plasma was stored at -80°C. Plasma cytokine and chemokine concentrations were quantified by multiplex protein arrays, according to the manufacturer’s instructions (BioRad Laboratories, Hercules, CA), and as described in [18]. Plasma levels of the following cytokines and chemokines were measured: IL-1 alpha, IL-1 beta, IL-2, IL-3, IL-4, IL-5, IL-6, IL-10, IL-12p40, IL-12p70, IL-13, IL-17, CCL11, granulocyte colony-stimulating factor (G-CSF), granulocyte macrophage colony-stimulating factor (GM-CSF), chemokine (C-X-C motif) ligand 1 (CXCL1, KC), CCL2 (MCP-1), CCL3 (MIP-1 alpha), CCL4 (MIP-1 beta), CCL5 (RANTES) and tumor necrosis factor alpha (TNF alpha).

To account for the hemodilution at the end of the procedure, all biochemistry and cytokine values were corrected according to the modified equation described by Roth-Isigkeit et al. [10] In short, values for clinical chemistry and cytokine concentrations were corrected for using the following formula:

Value(calculated)=(Value(measured)xHematocrit(measured))/Hematocrit(baseline)

### Histology

After termination of the experiments, internal organs (liver, kidneys, lung, and heart) were collected and fixed in 4% paraformaldehyde (PFA). Subsequently, tissues were stored for 24 h at 4°C and embedded in paraffin. Periodic acid-Schiff (PAS) staining was performed on 2 μm paraffin sections to assess renal and liver morphology. AKI was scored using a semi-quantitative grading system based on the amount of acute tubular necrosis seen upon histological evaluation. Both the tubuli in the cortex and in the outer medulla were scored separately. No signs of AKI = 0, focal AKI with < 10% of tubuli of the cortex affected = 1, mild AKI with 10–25% tubuli affected = 2, moderate AKI with 25–50% tubuli affected = 3 and severe AKI with >50% of the affected tubuli = 4. To assess liver damage, the loss of glycogen storage ability was evaluated. Glycogen storage defects appear pale in PAS while the normal morphology is characterized by violet hepatocytes corresponding to intracellular glycogen storage. To quantify Gr-1 infiltrates a scoring system from 0–3 was used: 0 –no infiltrates; 0.5 single GR-1 positive cells per view field (VF), 1-mild infiltration with 5–10 cells/VF; 2—moderate infiltration with 11–15 cells/VF; >16 cells/VF. For CD3 quantification the presence of focal accumulation of CD3 positive cells was scored with 1 or the absence with 0. Lung congestion was scored from 0 –no congestion, 1 –mild, 2- moderate, 3- severe congestion.

### Immunohistochemistry

Immunohistochemical staining was carried out on paraffin sections. After deparaffinization, histological sections were stained for CD3 positive T-lymphocytes (rabbit anti mouse 1:250, DAKO), monocytes/macrophages (F4/80; Biolegend, San Diego, CA, USA, rat anti-mouse, 1:200), neutrophils (Gr-1; AbD serotec, München, Germany, rat-anti mouse, 1:1000), alpha-1-microglobulin (A1M; donation from Dr. Grams Medical Centre Lund, Lund Sweden, rabbit anti-mouse, 1:500), and neutrophil gelatinase-associated lipocalin (NGAL; Dianova, Hamburg, Germany, rat anti-mouse, 1:1000). The incubation time of the primary antibodies was 60 min at room temperature. An Alexa Fluor 555-conjugated secondary antibody (Invitrogen, Carlsbad, California, USA, goat anti-rat IgG, goat anti-rabbit IgG, 1:500) for fluorescent visualization of bound primary antibodies was additionally incubated for 60 min in the dark. Analysis of the slides was done on a Leica DMLB microscope with a Leica DFC425C camera (Leica, Wetzlar, Germany). A1M was assessed by estimation of the percentage of A1M-expressing tubuli in the cortex indicating normal tubular transport function. NGAL is a marker of tubular damage and is upregulated upon AKI. The percentage of NGAL expressing tubuli was estimated in 10 different view fields at 200-fold magnification. The cell infiltration was scored as follows: 0 = no infiltrates, 1 = mild infiltration <10 cells per view field (VF), 2 = moderate 11–25 cells/VF, 3 = severe >26 cells/VF with multilayers of infiltrates in the interstitial space.

### Statistics

Statistical analysis was performed using GraphPad Prism version 5.0 software (GraphPad Software Inc., San Diego, CA, USA). Independent Student t-test was used to compare CBP and healthy controls. Significant differences were indicated as follows: *P < 0.*05*, **P < 0.*01*, ***P < 0.*001*. Data are presented as the mean ± standard deviation (SD).

## Results

### CPB with MHCA affects the liver and kidney function

Liver enzymes (ALT, AST) did not differ significantly between CPB and controls (Table 1). This indicates that there was no hepatic congestion due to right heart failure during the time on CPB. Lactate dehydrogenase (LDH) was significantly elevated in the CPB group, possibly resulting from hemolysis as blood is mechanically damaged in the CPB system. Hemolysis can aggravate renal damage because high concentrations of free hemoglobin can cause vasoconstriction and microcirculation impairment. Kidney function was indeed affected since relative serum creatinine and BUN were significantly increased in the CPB group compared to controls (Table 1).

**Table 1:** The effect of CPB on liver enzymes and kidney function.

	Control*	after CPB*	p-value
**ALT** (U/L)	138± 49	732± 348	n.s.
**AST** (U/L)	76± 14	328± 232	n.s.
**LDH** (U/L)	430± 72	7761± 1886	<0.01
**s-creatinine (**μmol/L)	24.46± 2.43	66.50± 8.81	<0.01
**BUN (**mmol/L)	9.01± 0.39	26.17± 3.44	<0.001

*Normalized liver and kidney function based on hematocrit after CPB in comparison with control animals.

n.s., not significant

n = 8

### Multi-organ injury after CPB with MHCA

**Fig 1:**
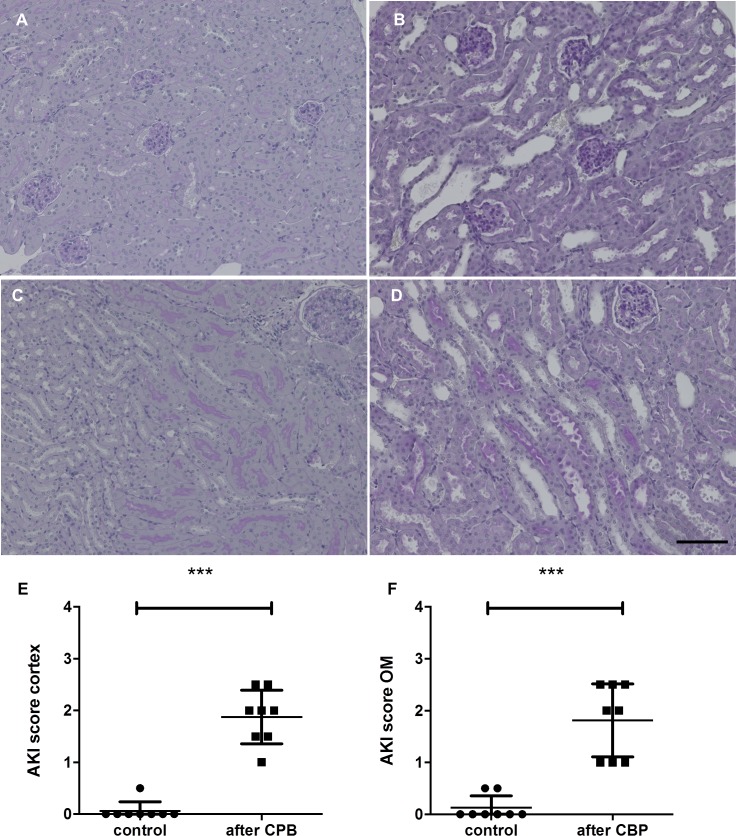
**PAS staining of the kidney after CPB with MHCA in (A, C) healthy control animals and (B, D) after CPB.** The upper row shows the cortex, the lower row the outer medulla. Kidney morphology after CPB showed acute tubular damage with tubular dilation. Semi-quantitative analysis was done for the cortex and outer medulla separately (E, F, ***P<0.*001*). Scale bars, 100 μm.

**Fig 2:**
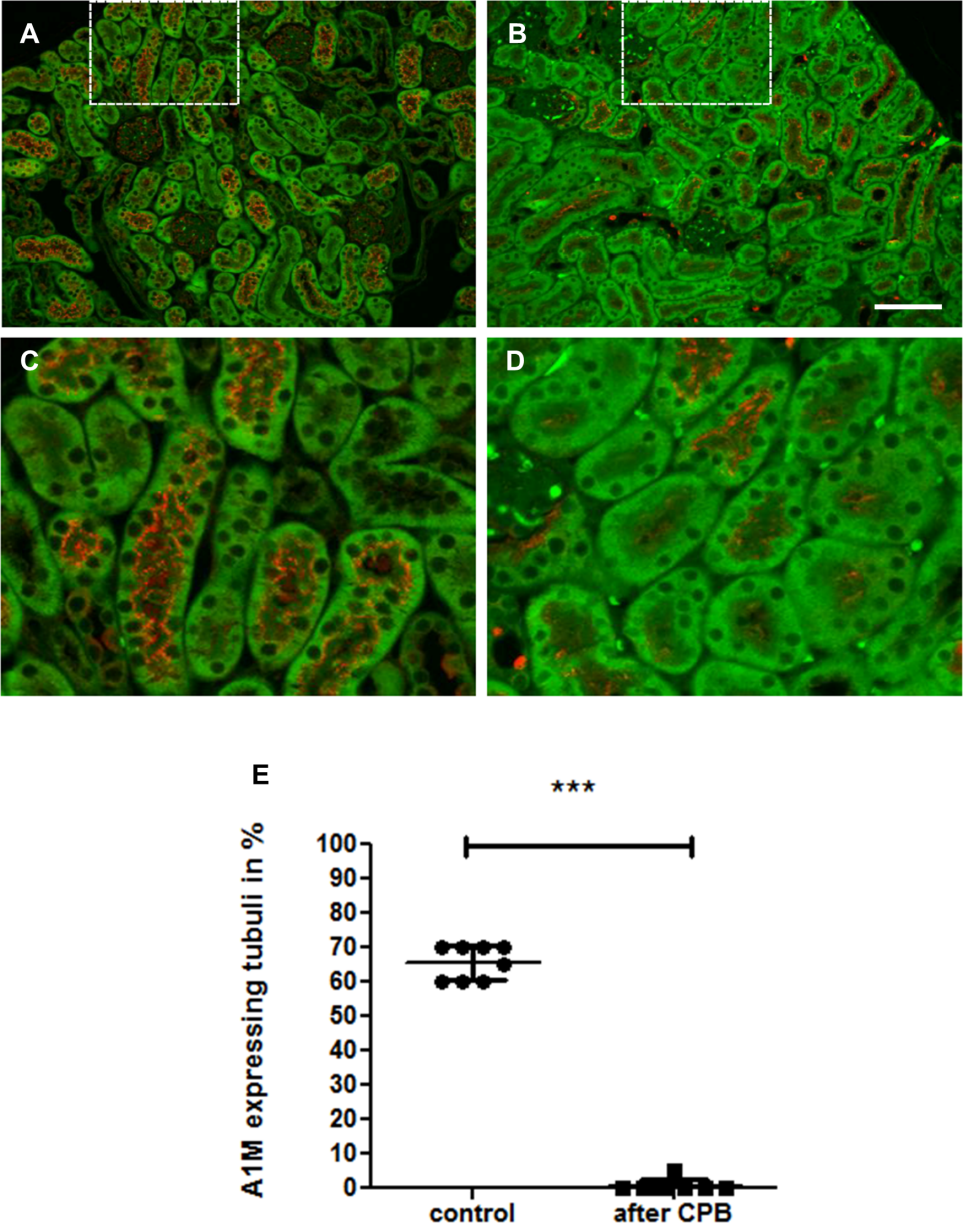
A1M immunofluorescence of the kidney as an indicator of tubular transport reveals that CBP results in total loss of tubular transport function. In healthy kidneys (A, C), 60–70% of tubuli are A1M positive (red), after CPB the percentage of normal tubuli is significantly decreased. (B, D). C and D are magnifications from the white squares above. Estimation of A1M-expressing tubuli is shown in E (***P<0.*001*) and reflects tubuli with normal tubular transport. The darker green areas are due to autofluorescence of kidney tissue. Scale bar, 100 μm.

Analysis of renal histology showed early signs of AKI with loss of brush border, tubular dilatation, vacuolization, and detachment of tubular epithelial cells (Fig 1). Staining for NGAL, a supposed biomarker of AKI [19], was negative in both groups most likely due to the early time point of organ retrieval 20 min after the end of surgery. Immunofluorescence for A1M, a marker of normal tubular transport function, showed a significant decrease of A1M-positive tubuli (Fig 2), indicating tubular dysfunction.

**Fig 3:**
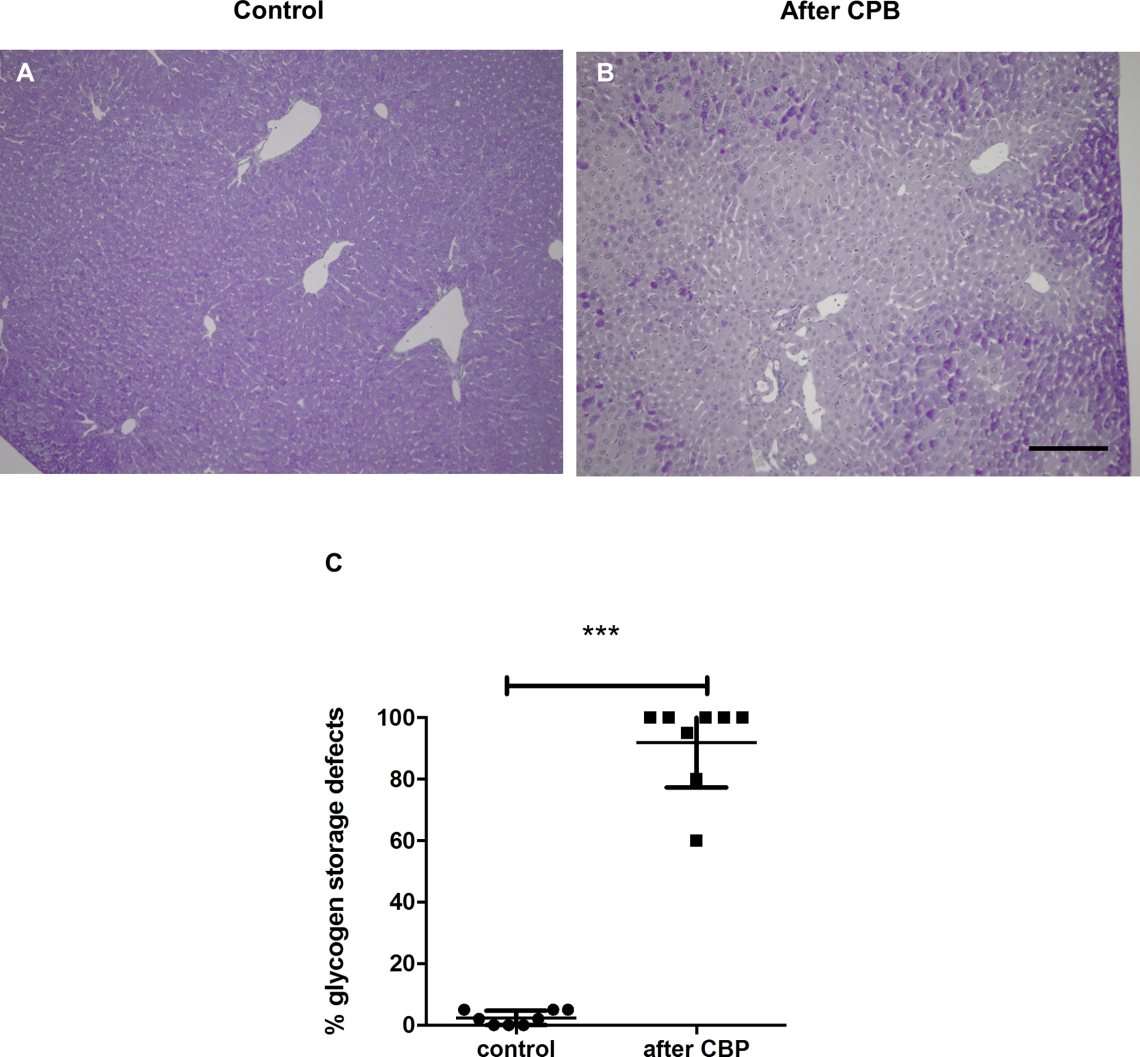
PAS staining of the liver reveals loss of glycogen storage capacity after CPB with MHCA. (A) Representative liver sections of healthy controls and (B) CBP/MHCA experimental mice. Dark purple staining indicates normal glycogen storage capacity and pale areas indicate loss of glycogen storage in injured hepatocytes. The proportion of hepatocytes demonstrating loss of glycogen storage capacity was quantified. CBP caused reduction of glycogen storage capacity (C, ***P<0.*001)*. Scale bar, 100 μm.

**Fig 4:**
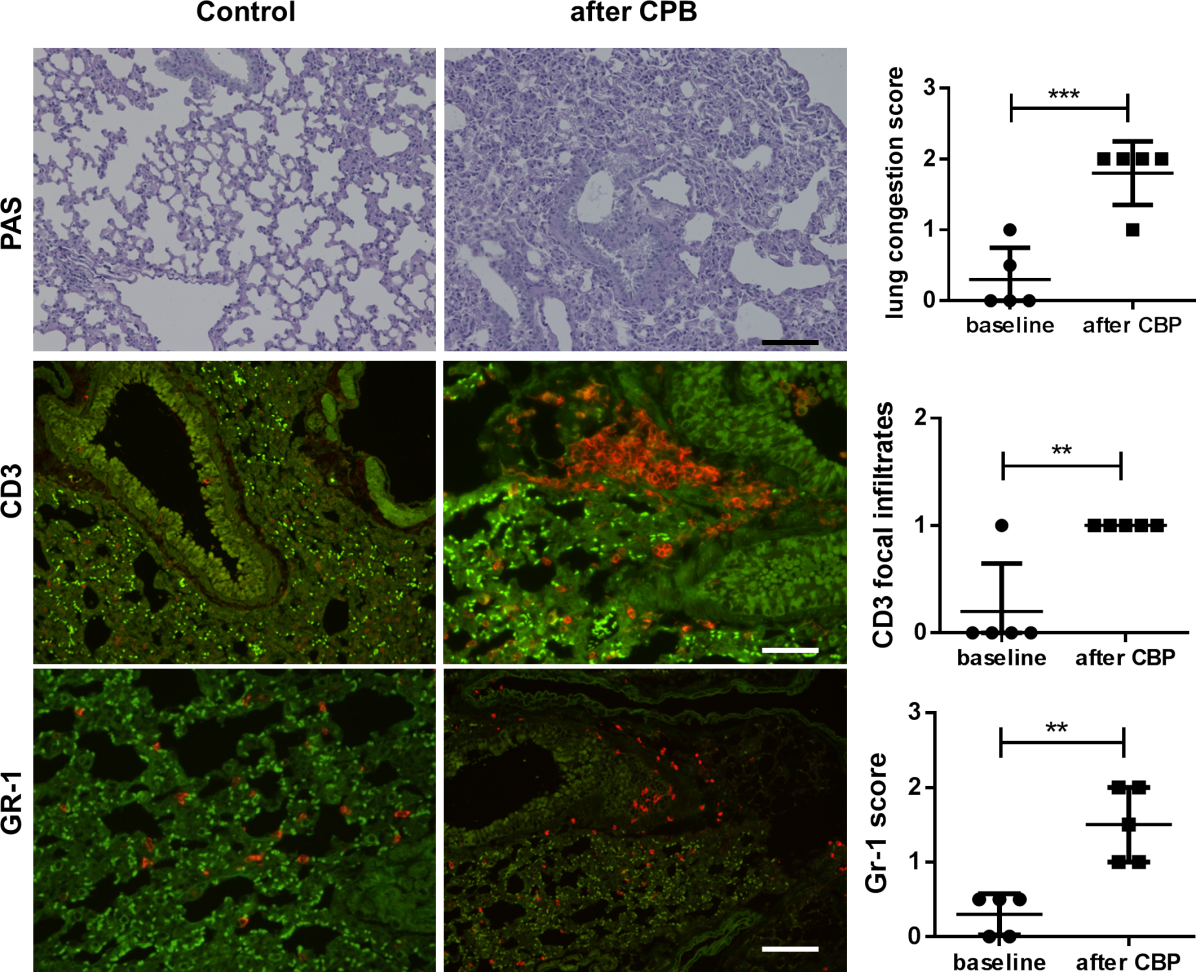
Lung histology and immunofluorescence of infiltrating leukocytes (red) revealed lung injury and inflammatory cell infiltration after CPB. Representative images of lung tissue from control animals (left row) and animals after CPB/MHCA (right row). (A-B) PAS staining showed pulmonary congestion, atelectasis, and an increase in cellular infiltrates. The scoring system was from 0–3 and revealed moderate congestion in all CPB/MHCA lungs. Immunofluorescence revealed enhanced (C, D) CD3+ T lymphocyte infiltration with focal accumulation after CBP/MHCA and enhanced diffuse (E, F) The presence of CD3 focal infiltrates was scored with 1, the absence with 0. GR-1+ neutrophil infiltration (red) showed that CPB/MHCA caused enhance neutrophile infiltration (scoring from 0–3). The bright green signals represent tracked red blood cells and the darker green areas are due to autofluorescence of lung tissue. Scale bar, 100 μm.

In the liver, PAS staining in hepatocytes is a sign of intact glycogen storage capacity while pale hepatocytes indicate reduced glycogen storage capacity. When the proportion of hepatocytes demonstrating loss of glycogen storage capacity was quantified, a significant increase in damaged liver tissue was observed after CPB (Fig 3). Lung histology revealed pulmonary congestion, atelectasis, and an increase in leukocyte infiltration after CPB (Fig 4). Immunofluorescent staining of the lungs showed an increase in focal CD3+ T-lymphocyte infiltration (Fig 4C and 4D) and diffuse enhancement of GR-1+ neutrophil infiltration (Fig 4E and 4F). An increase in F4/80-positive macrophages was not observed (Fig 4G and 4H). In the heart, there were no signs of myocardial damage, edema, or cellular infiltration in either the CPB or control groups.

### CPB elicits a rapid systemic cytokine and chemokine response

**Fig 5:**
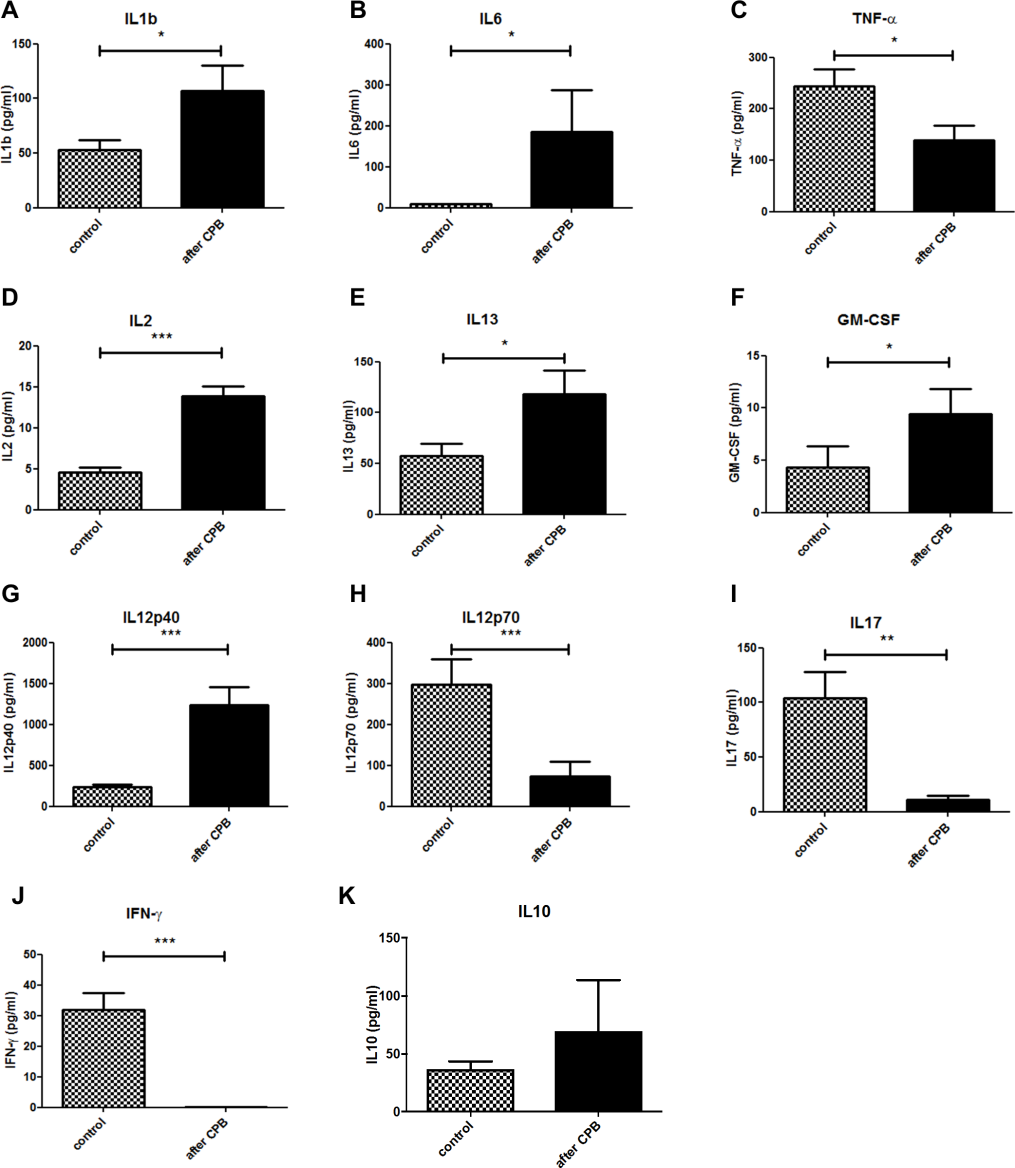
CPB elicits a systemic innate cytokine response. A significant increase in the concentration IL-1b (A) and IL-6 (B) in plasma was observed post-CPB compared to control. *P< 0*.*05*. n = 8 mice per group. Plasma concentrations of T-cell regulatory cytokines IL-2 (D), IL-13 (E), GM-CSF (F), IL12p40 (G),IL12p70 (H), IL-17 (I), IFNgamma (J) and IL-10 (K). All values were corrected for hemodilution.

**Fig 6:**
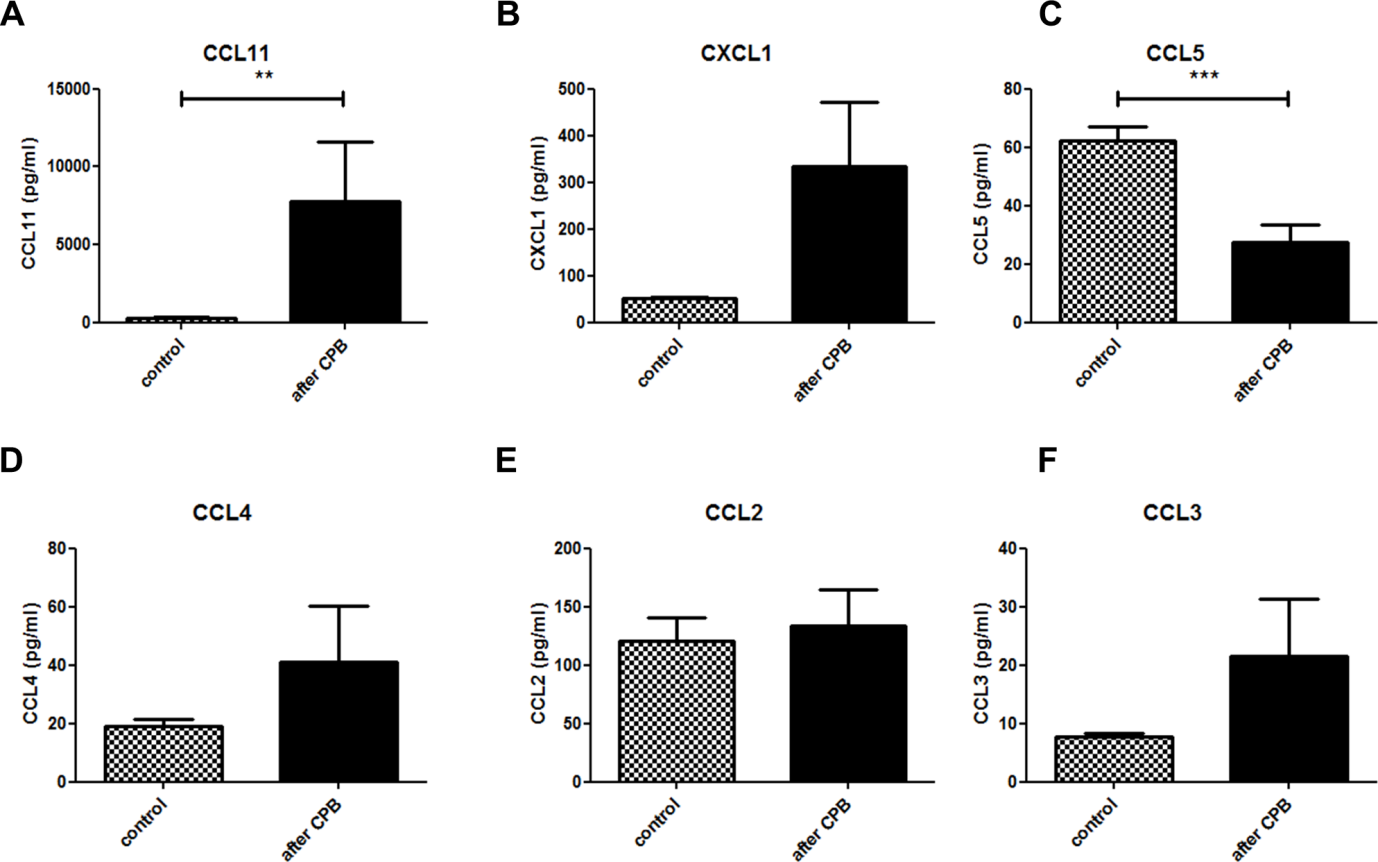
Specific systemic chemokines are induced by CPB. Expression of CCL11 (A) and CCL5 (C) showed a significant difference between the control and CPB group. CXCL1 (B) showed p = 0.07. There was no statistically significant difference seen for CCL4 (D), CCL2 (E), or CCL3 (F). All values were corrected for hemodilution.

Heparin plasma of control animals was used for the determination of systemic baseline cytokine and chemokine levels. The same correction for dilution was carried out as for clinical chemistry. Our results showed a significant release of several cytokines into the plasma post-CPB. A significant increase in two highly pro-inflammatory cytokines, IL-1beta and IL-6 (Fig 5A and 5B) was seen after CPB compared to control. Interestingly, TNF alpha levels were lower in the CPB group compared to the control group (Fig 5C). Furthermore, there was a significant increase in the concentration of IL-2 (Fig 5D), IL-13 (Fig 5E), GM-CSF (Fig 5F), and IL-12p40 (Fig 5G) suggesting a balance between a pro-inflammatory T helper cell (Th1) response via IL-2 and an anti-inflammatory Th2 response via IL-13 and IL-12p40, the common subunit of IL-12 and IL-23. In contrast, effector cytokines like the Th17 cytokine IL-17 (Fig 5I), and the Th1 cytokines IFNgamma (Fig 5J), and IL-12p70 (Fig 5H) were significantly lower in the control group compared to the CPB group. Other cytokines like IL-10 (Fig 5K) were not significantly altered arguing for a differential regulation of immune mediators. Since other cytokines (eg. IL-3, IL-4, and IL-5) were low and not significantly altered, this pattern indicates a rather Th2-dominated T cell response that suppresses Th1 and Th17 responses. A similar regulation was observed with respect to chemokines. Plasma concentrations of granulocyte-recruiting chemokines like CCL11 and CXCL1 (KC) were increased post-CPB compared to control although CXCL1 values were not statistically significant (p = 0.07) (Fig 6A and 6B). Of note, concentrations of macrophage-recruiting chemokines CCL5 and CCL4 were reduced although only CCL5 reached statistical significance (Fig 6C and 6D) while CCL2, and CCL3 were not significantly altered (Fig 6E and 6F). This chemokine pattern suggest that neutrophil granulocytes rather than monocytes may be activated and recruited which resembles the observation in immune histology (Fig 6).

## Discussion

The goal of this study was to investigate the relationship between acute organ injury and the expression of pro-inflammatory cytokines during CPB with MHCA. Using a small animal model of CPB with MHCA, we characterized the early events of organ damage using histology and immunohistochemistry.

### Early pathological signs of acute organ injury after CPB with MHCA

After CPB, there were signs of AKI even as early as 20 min after the end of the procedure. Compared to control animals, a significant increase in renal tubular damage and loss of tubular function was observed. Previous work by Tirilomis et al. [20] showed similar results after MHCA using a similar histological scoring system for kidney damage in neonatal piglets. Furthermore, an association between hemolysis, renal hypoxia, and acute kidney damage has been postulated [21–23]. Our data support these findings by detecting both significantly increased LDH values and histological tubular damage in the CPB group. Tubular function, as measured by A1M-positive tubular area, was strongly diminished in the CPB group. The renal damage was further indicated by the significant increase in serum creatinine and BUN after CPB. A1M is a heme-binding macroglobulin and a radical scavenger which is synthesized in most cells of the body, but mainly in the liver. It circulates in the blood and is filtered in the glomeruli and reabsorbed by proximal tubular epithelial cells. Reduced tubular A1M expression in the vesicles reflects a loss of energy supply and the breakdown of normal tubular transport function [24]. A1M has also been described as a urinary biomarker for renal diseases and previous work showed a strong association between elevated A1M urine concentrations and postoperative kidney failure in patients after CPB [25, 26]. Therefore, this data supports that our mouse model accurately models CPB-induced AKI. NGAL, another biomarker of acute kidney injury [19], was not significantly increased post-CPB [25,27]. It is likely that the short follow-up time (20 min) after the experimental procedure was too short to observe changes in NGAL expression which is up-regulated approximately 2–6 h after surgery [25].

Liver injury was also detected in our model and was indicated by a severe increase in liver enzymes (AST and ALT) due to hepatocyte damage. We observed loss of glycogen storage capacity in the liver post-CPB indicating a metabolic dysfunction with reduced glycogen storage capacity of the hepatocytes that may be associated with hypoxia of the liver during CPB. No morphological signs of early myocardial damage were seen post-CPB, however, this is not unexpected since myocardial edema usually develops as late as 24 hours postoperatively [28].

Following CPB, pulmonary changes were seen in form of structural alveolar changes and a slight increase in cellular infiltration. These results corroborate previous findings in a rat model of CPB [29,30]. Our findings did not show a significant increase in pulmonary macrophage infiltration, which is in concordance with previous work done by Francis et al. [31] who found a significant increase in macrophage infiltration as late as 24–48 h after induction of lung injury. In future studies, longer follow up is planned to compare the macrophage activation to previous studies.

### Adaptive and innate immune cell responses after CPB

CPB triggers an inflammatory response due to a variety of insults, eg. surgical trauma, blood contact with foreign surfaces, ischemia/reperfusion, and endotoxemia. Acute responses to stimuli are usually followed by the activation and migration of T cells, granulocytes, and monocytes/macrophages. In severe cases, systemic over-activation of the immune system can lead to SIRS with organ damage and death. Our data showed a differential regulation of the release of cytokines and chemokines involved in activation of innate and adaptive cellular immunity after CPB with MHCA.

T-lymphocytes were shown to be activated during CPB and to induce pulmonary damage [32]. Our CPB group showed elevated plasma concentrations of cytokines of both Th1 (e.g. IL-2, GM-CSF, and IL-12p40) and the Th2 (IL-13) groups suggesting activation of T helper lymphocytes. The significant decrease of TNFalpha, IFN gamma, IL-17, and IL-12p40 plasma concentrations indicates a shift towards a Th2 response that may limit cytotoxic Th1 and Th17 responses. Histological findings, however, did not show a significant increase in T lymphocyte infiltration into lung tissue likely due to the early time point chosen for analysis. Thus, a systemic Th2-dominated response may precede T cell infiltration into the organs−which is supported by the chemokine data. In rats undergoing CPB with MHCA, initial leukocyte counts are low due to the hemodilution effect of the priming volume, yet early activation of cellular immunity due to elevated cytokine concentrations has been observed [33]. A significant increase in IL-2 expression does not only indicate T cell activation, it can also suggest a Th1/Th2 shift, defined as an increase in Th2 cell activity and a delayed recovery of Th1 cell function. A Th1/Th2 shift is believed to be responsible for the reduced competence of the specific and non-specific immune response in patients after CPB [34].

### Chemokine release after CPB

Chemokines are a family of chemotactic cytokines that stimulate the recruitment of leukocytes into tissues and maintain their circulatory homeostasis. In our analysis of chemokines circulating in the blood early after CPB, we observed increased levels of CCL11 and CXCL1, chemokines linked to granulocyte recruitment [35,36]. In our histology analysis, we observed a small increase in neutrophil infiltration in the lungs after CPB. Research done by Zhang et al. [37] showed significant neutrophil infiltration occurs only after 12 h after CPB.

In contrast, CCL5 (RANTES), a chemokine involved in ischemia-reperfusion injury and associated with lymphocyte and monocyte recruitment [38,39], was lower in the CPB group in comparison to control. Patient studies in have shown CCL5 concentration decreases during CPB and return to normal values one hour after termination of CPB [40,41]. Our data showed a similar drop in CCL5 concentration but since our blood samples were collected 20 minutes after the procedure, we postulate that CCL5 concentrations were not yet fully restored to baseline levels. Also, as previous work showed low levels of CCL5 to be associated with low lymphocyte counts [42], this may also explain the low lymphocyte count in our lung tissues. Furthermore, treatment with anti-CCL5 after myocardial infarction results in reduced neutrophil and macrophage infiltration and smaller infarction size [43]. Thus, down-regulation of consumption of CCL5 during CPB seems to be protective of organ damage due to interference with tissue / circulatory homeostasis of myeloid cells.

The potent monocyte and macrophage chemoattractants CCL3 and CCL4 have been associated with the initiation of CPB and MHCA in patients[44]. Of note, we observed a non-significant increase at very low levels for CCL3 and CCL4 and even stable CCL2 concentrations in blood after CPB, which indicates that at least these chemokines may not play an important role of macrophage recruitment and activation. Thus, CXC rather than CC chemokines may contribute to the monocyte/macrophage infiltration into tissues in this early kinetic.

The study has some limitations: first, the follow-up period is rather short and future studies should address the effects after CBP at later time points such as 24 hours since leukocyte infiltration may require more time. Second, leukocyte subsets were stained by immunohistochemistry, but to further characterize leukocyte function, flow cytometry would be helpful to distinguish between the activation status of the cells. Third, differential white blood cell count was not done thus the quantification of leukocyte subsets in the peripheral blood cannot be correlated with tissue infiltration of leukocytes. This would be interesting to do in follow up studies. Future studies will address these important aspects.

## Conclusions

Overall, we could demonstrate an increase in Th2 cytokine expression and correlate this to histological damage seen early after CPB with MHCA. Further investigation of the cytokine network and histological changes at later time points after termination of CPB is needed to determine the full effect of pro-inflammatory cytokine expression on the immunological response to CPB with MHCA. Evaluation of early cytokine expression could contribute to the prognostic assessment of potential multi-organ damage. Furthermore, it provides the possibility to establish organ protection strategies after circulatory arrest. A number of studies have been published on minimizing the damage of CPB and cardiac arrest on internal organs [45–57]. Cytokine measurements in prolonged CPB and moderate circulatory arrest allows the evaluation of immunological involvement and its effect on CPB-induced organ damage. Further research must be done to identify specific and systemic effects of defined cytokines on target organs in terms of the pathomechanisms of SIRS under CPB. Furthermore, as a next step, we will use our model to study whether cytokine adsorption during CPB can prevent organ damage.
